# Investigation of deep learning model for predicting immune checkpoint inhibitor treatment efficacy on contrast-enhanced computed tomography images of hepatocellular carcinoma

**DOI:** 10.1038/s41598-024-57078-y

**Published:** 2024-03-19

**Authors:** Yasuhiko Nakao, Takahito Nishihara, Ryu Sasaki, Masanori Fukushima, Satoshi Miuma, Hisamitsu Miyaaki, Yuko Akazawa, Kazuhiko Nakao

**Affiliations:** 1 Department of Gastroenterology and Hepatology, Nagasaki University Graduate School of Biomedical Sciences, 1-7-1 Sakamoto, Nagasaki City, Nagasaki Japan; 2 Department of Gastroenterology and Hepatology, Isahaya General Hospital, 24-1 Eishohigashimachi, Isahaya, Nagasaki Japan; 3 Department of Histology and Cell Biology, Nagasaki University Graduate School of Biomedical Sciences, 1-12-4 Sakamoto, Nagasaki City, Nagasaki Japan

**Keywords:** AI, HCC, YOLO, CNN, CAMs, Cancer, Computational biology and bioinformatics, Gastroenterology

## Abstract

Although the use of immune checkpoint inhibitors (ICIs)-targeted agents for unresectable hepatocellular carcinoma (HCC) is promising, individual response variability exists. Therefore, we developed an artificial intelligence (AI)-based model to predict treatment efficacy using pre-ICIs contrast-enhanced computed tomography (CT) imaging characteristics. We evaluated the efficacy of atezolizumab and bevacizumab in 43 patients at the Nagasaki University Hospital from 2020 to 2022 using the modified Response Evaluation Criteria in Solid Tumors. A total of 197 Progressive Disease (PD), 271 Partial Response (PR), and 342 Stable Disease (SD) contrast CT images of HCC were used for training. We used ResNet-18 as the Convolutional Neural Network (CNN) model and YOLOv5, YOLOv7, YOLOv8 as the You Only Look Once (YOLO) model with precision-recall curves and class activation maps (CAMs) for diagnostic performance evaluation and model interpretation, respectively. The 3D t-distributed Stochastic Neighbor Embedding was used for image feature analysis. The YOLOv7 model demonstrated Precision 53.7%, Recall 100%, F1 score 69.8%, mAP@0.5 99.5% for PD, providing accurate and clinically versatile predictions by identifying decisive points. The ResNet-18 model had Precision 100% and Recall 100% for PD. However, the CAMs sites did not align with the tumors, suggesting the CNN model is not predicting that a given CT slice is PD, PR, or SD, but that it accurately predicts Individual Patient's CT slices. Preparing substantial training data for tumor drug effect prediction models is challenging compared to general tumor diagnosis models; hence, large-scale validation using an efficient YOLO model is warranted.

## Introduction

Hepatocellular carcinoma (HCC) is the most common primary liver cancer worldwide and a considerable cause of cancer-related deaths. The use of immune checkpoint inhibitors (ICIs) has recently shown promising outcomes in HCC treatment^1,2^. Additionally, there has been significant focus on integrating medical imaging with predictive models based on AI to improve patient management and treatment outcomes^3^.

CT plays a crucial role in diagnosing, staging, and monitoring HCC^4^. However, no substantial dataset of CT images combined with clinical and outcome data has yet been used to develop AI prediction models for HCC prognosis. The integration of AI with HCC prognosis offers numerous advantages. For example, it can help identify patients who are highly likely to respond to ICIs therapy in the early stages. Furthermore, AI-predictive models can assess the efficacy of treatment and identify patients who require alternative interventions or therapies. This leads to an opportunity to promptly modify treatment plans and contributes to improved outcomes.

Deep learning techniques in the field of hepatocellular carcinoma have been used for lesion detection, liver tumor segmentation^5–10^, and differential diagnosis^11^. However, although outcome prediction after transarterial chemoembolization (TACE) treatment for liver cancer has been reported^12–18^, there are no reports on outcome prediction after ICIs treatment.

Therefore, we trained a deep convolutional neural network (CNN) and a You Only Look Once (YOLO) model to predict the outcome of ICIs treatment. Additionally, we utilized the CAMs algorithm to identify the relevant morphological features for machine learning predictions and locate image features that the CNN associates with HCC treatment efficacy. As a result, we gain insight into the specific parts of the image the CNN examines to determine treatment efficacy.

In a previous report, ResNet-18, a CNN model, was found to be effective in predicting microvascular invasion using preoperative CT images of hepatocellular carcinoma (HCC)^19^. In contrast, the YOLO model, which is gaining popularity in object detection, enables real-time discrimination and localization of objects. However, no studies have yet been conducted on the efficacy of liver cancer treatment using the YOLO model.

Our research aims to investigate the potential of AI in supporting physicians with treatment decisions and its future usability. In this study, multiple images of each patient were used to predict the prognosis of liver cancer accurately. Many studies have shown that background liver status, including hepatic spare ability and Child–Pugh score, can be a crucial indicator. Interestingly, it is not uncommon to observe different types of malignant liver cancer concurrently in a single patient during clinical practice. The YOLO model shows promise in precisely predicting the various states of liver cancer and is expected to aid physicians in making informed decisions regarding treatment.

One of the most cited issues in AI development for medical use is the lack of adequate data sets. With numerous anticancer treatments available and countless new drugs introduced regularly, it is difficult to obtain enough image and data sets to validate a drug's efficacy, even in cases of common cancers. In real-world clinical practice, there is a necessity to perform exploratory studies that can develop models to precisely determine drug effectiveness from a restricted number of tumor images and treatment efficacy data. Our report details the development of an AI-based model that predicts the effectiveness of liver cancer treatment by recognizing contrast-enhanced CT scan features before the introduction of ICIs.

## Methods

### Patients

From 2020 to November 2022, there were 43 (except duplicate cases) patients with unresectable HCC who underwent CT 3 months prior to treatment initiation. The median age of the sample was 72 years. In addition, they were administered atezolizumab plus bevacizumab therapy at Nagasaki University Hospital (Table 1). This study included 43 patients allocated to the atezolizumab plus bevacizumab group. Patients with administration period of less than 3 weeks and in whom treatment response could not be determined were excluded from the study.

**Table 1:** Clinical characteristics of the patients.

Factor		(n = 43)
Age	Years	72.0 (48–88)
Sex	Male/female	33/10
BMI	kg/m^2^	22.2 (16.9–28.6)
Performance status	0/1/2/3	27/12/3/1
Child–Pugh class	A/B	40/8
Tumor size	cm	5.8 (1.0–19.0)
Macroscopic PV invasion	Vp3/4	7 (16.2%)
Extrahepatic spread	+	18 (41.8%)
BCLC stage	B/C	18/25
Etiology	B/C/NBNC	8/10/25
Plt	×10^4^/µL	16.7 (6.7–45.0)
T.bil	mg/dL	0.80 (0.3–2.0)
Alb	g/dL	3.50 (2.2–5.4)
ALT	IU/mL	26.0 (13–119)
AFP	ng/mL	90.9 (2–484,000)
DCP	mAU/mL	3016.0 (14–339,137)

AFP, Alpha fetoprotein; Alb, albumin; ALT, alanin aminotransferase; BCLC, balcelona clinic liver cancer; BMI, body mass index; DCP, des-γ-carboxy prothrombin; NBNC, nonBnonC; Plt, platelets; PV, portal vein; T. bil, Tortal bilirubin.

### Treatment protocol, evaluation criteria for response, and follow up of HCC

Intravenous atezolizumab (at a dose of 1200 mg) and bevacizumab (at a dose of 15 mg/kg of body weight) were administered to the patients every 3 weeks. The treatment was discontinued if unacceptable adverse events or clinical tumor progression were observed. Treatment response was evaluated using contrast-enhanced CT or MRI with the modified Response Evaluation Criteria in Solid Tumors^20^ every 8–12 weeks. The best response was adopted as the therapeutic effect.

### CT scanning protocols

HCC was evaluated by CT examinations by using one of three CT systems (Aquilion ONE and Aquilion Precision, Canon Medical Systems, Otawara, Japan; Somatom Definition Flash, Siemens, Erlangen, Germany). The scanning protocol was a rotation time of 0.5 s, beam collimation of 80 × 0.5 mm, reconstruction section thickness and interval of 1.0 mm, pitch factor of 0.6–1.2, table movement of 65 mm/s, default field of view (FOV) of 40 cm, 120 kV, and automatic exposure control. Nonionic contrast medium (100 mL of Omnipaque 240, Daiichi‐Sankyo, Tokyo, Japan; 100 mL of Oypalomin 300 and 65 mL of Oypalomin 370, Konica Minolta, Tokyo, Japan) at a dose of 520–600 mgI/kg was used for CT examination in 48 patients. The arterial, portal, and delayed phase images were scanned with the following delays: arterial phase, using a bolus tracking system (threshold attenuation of 200 Hounsfield units in the descending aorta at the level of the diaphragm; portal phase, 40 s after arterial phase; and delayed phase, 180 s after the beginning of contrast agent injection).

### Ethical considerations

This study was conducted in accordance with the 1964 Declaration of Helsinki and institutional and national ethical standards of the committees responsible for human experimentation. Informed consent was obtained from all the patients prior to the study. The study protocol was approved by the Ethics Committee of Nagasaki University Hospital (approval date, April 16, 2019; approval number, 19041523-4).

### Deep learning implementation and statistical analysis

ResNet-18^21^, a Convolutional Neural Network (CNN) model, and YOLOv5, YOLOv7^22^, YOLOv8 a You Only Look Once (YOLO) model, were used for supervised learning to predict treatment effects. Precision, Recall, F1 Score, Mean Average Precision (mAP) are the metrics that were used to evaluate the detection performance of the models. The Recall value describes the sensitivity of the object detection model. The F1 Score is the harmonic mean between the precision and the recall value. The mAP value represents the accuracy of the object detection model and is computed by calculating the area under the precision-recall curve. Class activation maps (CAMs)^23^ were employed to interpret the CNN models. Principal component analysis and 3D t-distributed Stochastic Neighbor Embedding (3D-tSNE)^24^ were used for feature analysis of the entire image. To validate the prediction model, 20% of the total data was used as the validation dataset. All training procedures were performed using NVIDIA A6000. The CNN model was implemented based on PyTorch.

## Results

### CNN model training strategy

**Figure 1:**
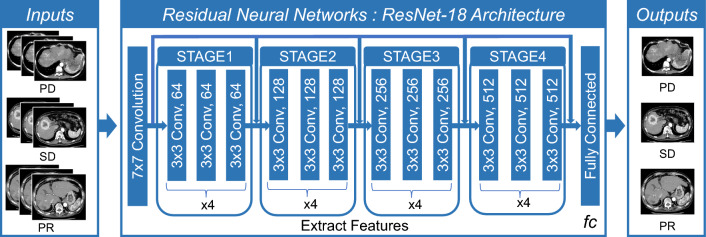
The architecture of the ResNet-18 convolutional neural network.

**Figure 2:**
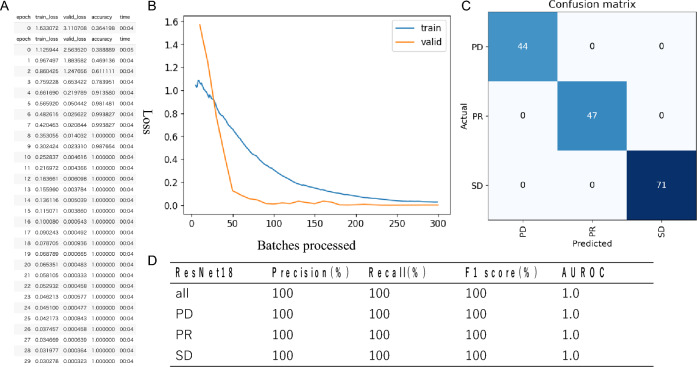
(**A**) and (**B**) show the process of CNN model training by Pytorch, whereas (**C**) depicts the confusion matrix of prediction results from 20% test datasets. CNN, Convolutional neural network.

**Figure 3:**
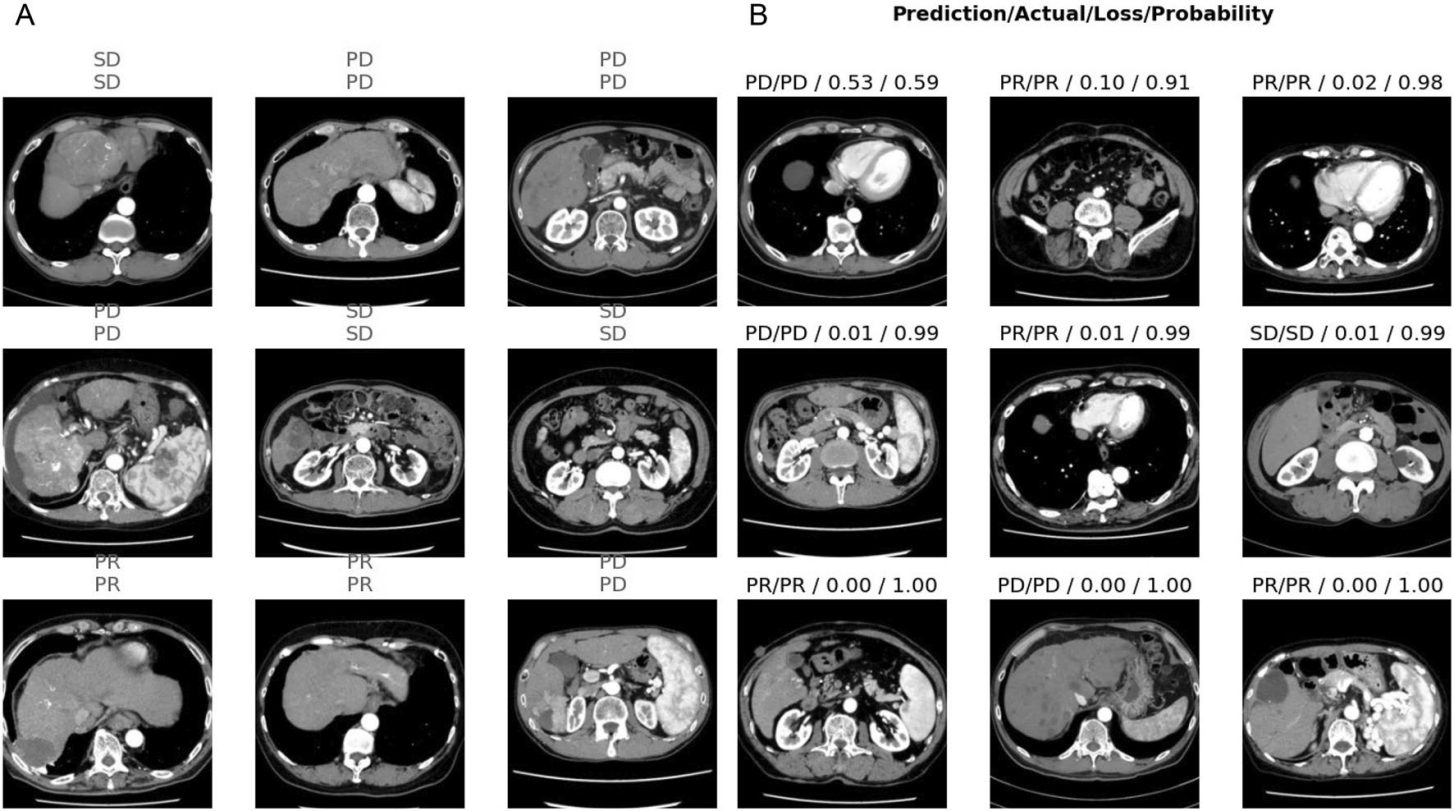
(**A**) Top label shows actual label second label shows the prediction model of each CT slices by ResNet-18, while (**B**) shows the computed loss and probability of each CT slices. CT, Computed tomography.

**Figure 4:**
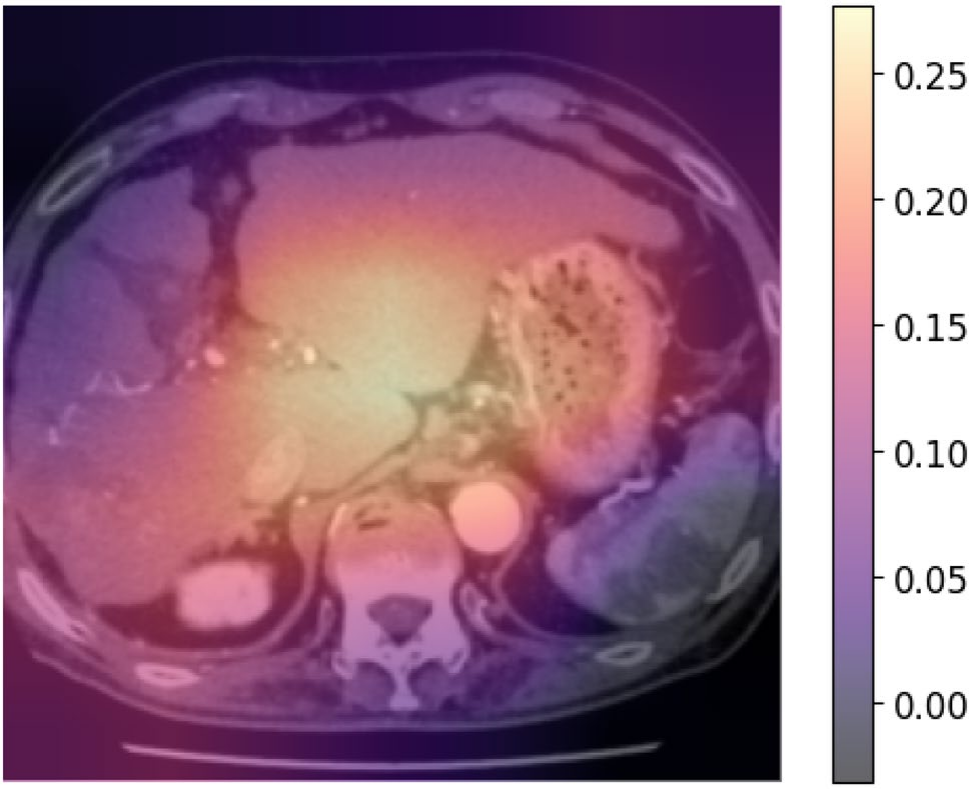
CAMs analysis with heatmap. The color bar to the right quantifies intensity of CAMs prediction site.

**Figure 5:**
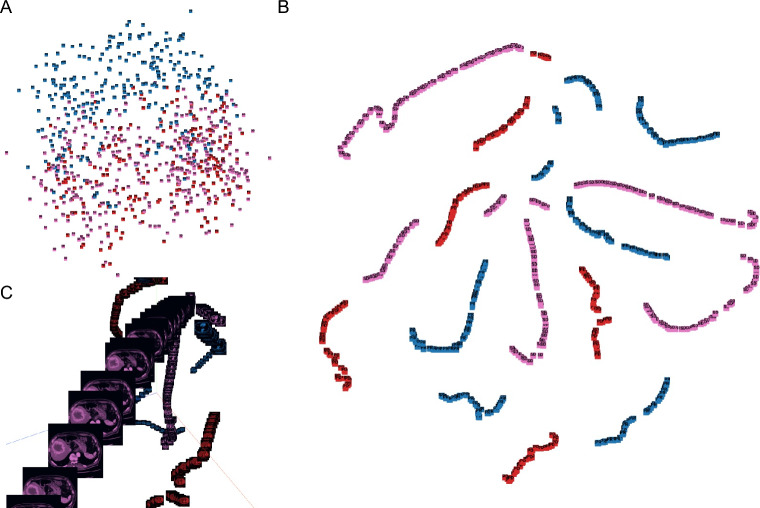
(**A**) Principal component analysis was performed on Tensorboad projector 3D-tSNE software. Red color represents PD. Blue color represents PR. Pink color represents SD. (**B**) tSNE analysis by 3D-tSNE. (**C**) shows higher magnificent of 3D-tSNE analysis. Each picture cluster represents CT slices with same patients. CAMs, Class activation maps; CNN, convolutional neural network; PCA, principal component analysis; 3D-tSNE, 3D t-distributed stochastic neighbor embedding.

We implement and optimize our deep convolutional neural network using fastai^25^, which is built on PyTorch. All choices of CNN hyperparameters or tweaks have been empirically tuned in order to optimize training. The cross-entropy loss is used to evaluate for multi-class classification tasks. We use the ResNet-18 of CNN architectures, which are enhanced versions of the original residual neural networks^21^. The ResNet-18 architecture is depicted in Fig. 1, with the initial layers accepting an input of an image with dimensions of 224 × 224 pixels and three RGB channels. We conducted the initial training using 80% of the available images from the 43 cases, totaling 810 images. This training was performed 30 epochs and observed that both the training loss and validation loss had plateaued, as shown in Fig. 2A and B. The model's classification output was based on the element with the highest probability in the output vector. The validation results using the designated dataset are presented in a confusion matrix format in Fig. 2C. Figure 2C and D showed the ResNet18 model had Precision 100% and Recall 100% for PD. Figure 3A shows a comparison between the actual predictions and ground truth labels. Figure 3B illustrates a slice with a high loss, denoting the magnitude of the difference between the correct and predicted values by the model. The interpretation regions of the ResNet18 model were visualized using CAMs (as shown in Fig. 4). The CAMs heatmap marked certain areas in red that did not correspond to tumor regions. Subsequently, comprehensive feature analysis of the complete dataset was performed using principal component analysis (Fig. 5A). Figure 5B and C illustrate that the 3D-tSNE analysis revealed data points tending to cluster for each individual case. 3D-tSNE showed that the conventional CNN model tended to concentrate on the specific features of each patient's internal organs, such as the background liver, instead of capturing the essential characteristics of HCC.

### YOLO model training strategy

**Figure 6:**
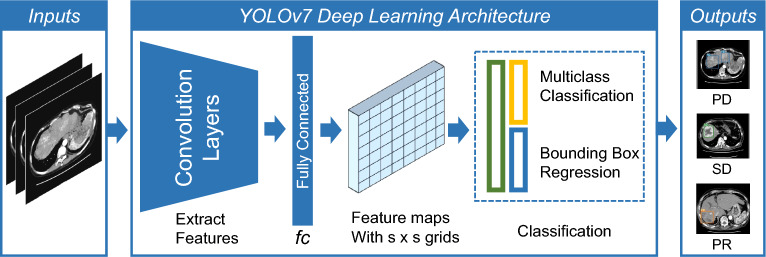
The architecture of the YOLO model. YOLO, You only look once.

**Figure 7:**
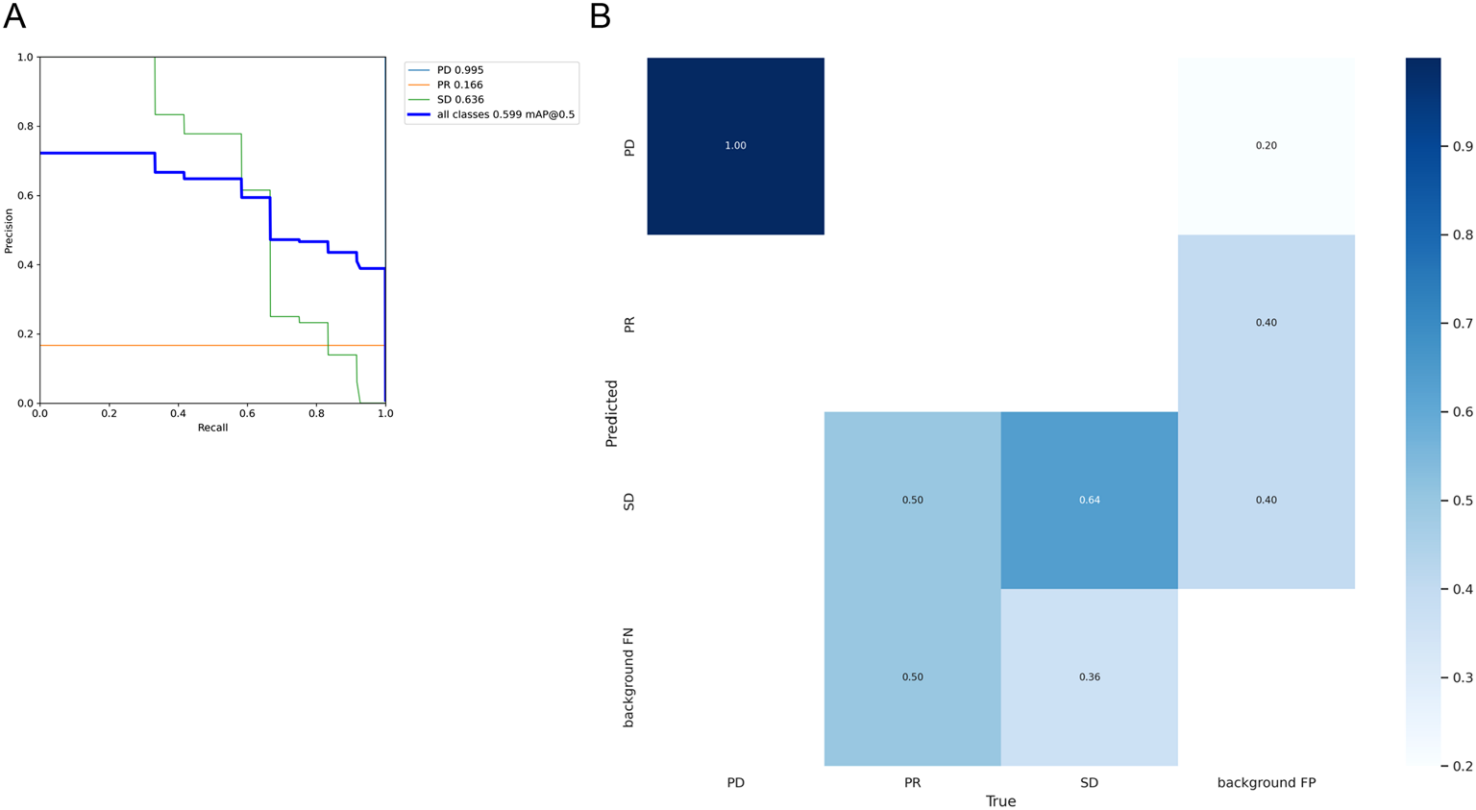
(**A**) Precision-recall curve for each prediction. Light blue color shows PD, Orange color shows PR, Green color shows SD, Blue color shows all the classes. (**B**) Depicts the confusion matrix of the YOLO prediction. YOLO, You only look once.

**Figure 8:**
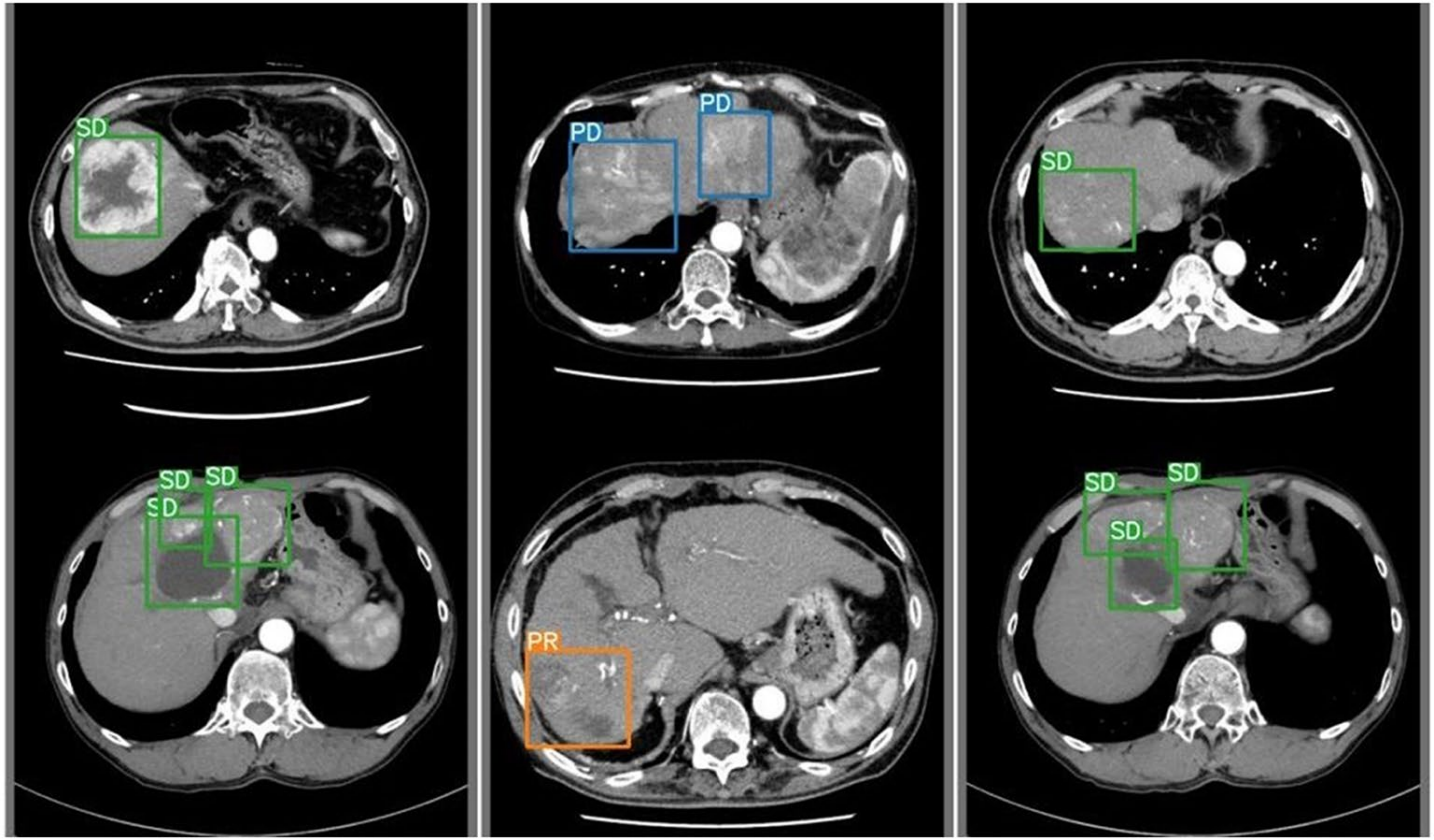
Six examples for the YOLO model predictions. YOLO, You only look once.

We have optimized and implemented our deep convolutional neural network utilizing the newest versions of the YOLO (You Only Look Once) framework, such as YOLOv5, YOLOv7, and YOLOv8, which are all based on the Darknet architecture and have been modified for increased accuracy and performance. Each iteration of the YOLO model has undergone meticulous configuration, refining hyperparameters and structural tweaks through empirical methods to optimize training outcomes. The loss function utilized in these models is an advanced variant of the original YOLO loss, which includes separate components for bounding box regression, objectness prediction, and class probability estimation. The network architectures for YOLOv5, YOLOv7, and YOLOv8 are showcased in Fig. 6. Each input layer in the design processes images that meet the specific dimensions of the models while maintaining three RGB channels. To ensure accuracy and reliability, our training regimen drew from an extensive range of classes, utilizing 80% of the available images and producing a robust dataset. Our training ran for 100 epochs. Table 2 was a summary comparison of the results for YOLOv5, YOLOv7, and YOLOv8 model in this dataset. YOLOv7 performed well in all categories, so we selected it for verification. YOLO model was designed to target the HCC region (Fig. 6). An analysis based on the YOLO model, as illustrated in Fig. 7, resulted in Precision 53.7%, Recall 100%, F1 score 69.8%, mAP@0.5 99.5% for PD (Table 3). Finally, Fig. 8 shows the predictive outcomes. This highlights the accurate localization of tumor regions and corresponding predictions of treatment efficacy.

**Table 2:** HCC treatment effect prediction comparison of YOLOv5, YOLOv7, and YOLOv8.

Model	Precision (%)	Recall (%)	F1 score (%)	mAP@0.5 (%)
YOLOv5	78.3	31.9	45.3	31.9
YOLOv7	98.3	44.4	61.1	58.4
YOLOv8	62.2	11.1	18.8	9.1

**Table 3:** HCC treatment effect prediction summary by YOLOv7.

YOLOv7	Precision (%)	Recall (%)	F1 score (%)	mAP@0.5 (%)
All	98.3	44.4	61.1	58.4
PD	53.7	100	69.8	99.5
PR	16.6	100	28.4	16.6
SD	58.8	58.3	58.5	63.6

## Discussion

The evolution of deep learning has resulted in significant progress in medical imaging, particularly in the segmentation of liver cancer using CT images^26^. Despite the widespread use of segmentation models, a gap remains in the development of models specifically designed for treatment selection. The primary findings of our study are supported by the differentiation between the existing models and what is urgently required in clinical settings.

The aim of our research is to explore the potential of AI in assisting physicians with treatment decisions and its future usability. The analysis of the image features in the liver and liver cancer-containing slices of patient reveals that all patient slices are clustered according to their respective slice numbers (Fig. 5C). This describes a characteristic of CT sequential images and how CNN model can learn image features that approximate slice features of different patients, regardless of the presence or absence of hepatocellular carcinoma information. It also explains how it is relatively easy to determine which slice belongs to which patient. In other words, CNN model is not predicting that a given CT slice is PD, PR, or SD, but that it accurately predicts individual Patient’s CT slices. However, this labeling does not serve our goal of identifying tumor therapeutic features. Despite the 100% accuracy, we chose to use YOLO model instead of CNN model to prevent overestimation and underestimation in future medical AI implementations. This issue could also arise in the development of medical AI for other types of medical images.

The YOLO model^22^ provided improved accuracy and a twofold clinical advantage. First, it accurately identified regions that play crucial roles in the decision-making process. Second, it introduces versatility, a crucial trait that is often overlooked in clinical settings. Identifying the decision basis is crucial, especially when considering imaging biomarkers such as pathological validation. The practical value of AI is maximized when physicians comprehend and have conviction in its decision-making process, highlighting the significance of transparency in AI prediction^27^.

Current paradigms for medical decision making are multifaceted. Doctors manage abundant data ranging from the general condition of the patient and performance status to complicated blood biochemistry and tumor markers information before determining a treatment modality. The major future challenge is to translate this intricate decision-making process smoothly into AI systems. How can AI integrate and operate with a comparable range of data as effectively as experienced physicians? We believe that the solution lies in models such as YOLO, which concentrate information in advance on relevant sections, such as HCC sites.

However, certain challenges remain. Preparing large quantities of accurately labelled data to predict tumor drug efficacy requires more intricate procedures than general tumor diagnostic models. The inherent efficiency of the YOLO model makes it a promising tool for large-scale validation applications.

Emulating the complex neural network of an experienced physician's brain requires a multimodal approach^28^. An ideal model would combine the relevant CT data extracted using the YOLO model with biological indicators, such as blood sample data. Such fusion could enable AI systems to replicate, and perhaps even improve, human diagnostic and predictive abilities in the oncology field.

## Data Availability

The datasets generated and analyzed during the current study are available in the predict ICIs-HCC repository, https://github.com/yasunakao/predictICI-HCCmodel.

## Abbreviations

3D-tSNE: 3D t-distributed stochastic neighbor embedding
CAMs: Class activation maps
CNN: Convolutional neural network
ICIs: Immune checkpoint inhibitors
YOLO: You only look once
